# Comparing visual inspection methods for parenteral products in hospital pharmacy: between reliability, cost, and operator formation considerations

**DOI:** 10.1136/ejhpharm-2024-004143

**Published:** 2024-05-24

**Authors:** Alexandre Jambon, Marie Forat, Chloé Marchand, Corinne Morel, Camille Merienne, Samira Filali, Fabrice Pirot

**Affiliations:** 1 Service Pharmaceutique, Plateforme FRIPHARM, Groupement Hospitalier Centre, Hôpital Edouard Herriot, Place d’Arsonval, Hospices Civils de Lyon, Lyon, Auvergne-Rhône-Alpes, France; 2 Service Biomédical, Groupement Hospitalier Centre, Hôpital Edouard Herriot, Place d’Arsonval, Hospices Civils de Lyon, Lyon, Auvergne-Rhône-Alpes, France; 3 Laboratoire de Recherche et Développement de Pharmacie Galénique Industrielle, UMR 5305, Plateforme Fripharm, Faculté de Pharmacie, UCBL, Lyon, Auvergne-Rhône-Alpes, France

**Keywords:** Drug Compounding, Environment, Controlled, Equipment Design, PHARMACEUTICAL PREPARATIONS, Pharmacopoeia, PHARMACY SERVICE, HOSPITAL, Quality Assurance, Health Care, Safety

## Abstract

**Introduction:**

The COVID-19 pandemic has led to unforeseen and novel manifestations, as illustrated by the management of drug shortages through the development of hospital production of sterile pharmaceutical preparations (P2S). Visual inspection of P2S is a release control whose methods are described in monographs of the European Pharmacopoeia (2.9.20) and the United States Pharmacopeia (1790). However, these non-automated visual methods require training and proficiency testing of personnel. The main objective of this work was to compare the reliability and speed of analysis of two visual methods and an automated method for detecting visible particles by image analysis in P2S. Furthermore, these methods were used to evaluate sources of particulate contamination during pre-production processes (washing, disinfection, depyrogenation) and production (filling, capping).

**Materials and methods:**

Three pharmacy technicians examined 41 clear glass vials of type I, 10 and/or 50 mL through manual visual inspection (MVI), semi-automated (SAVI), and automated (AVI) inspection. The vials were distributed as follows: (i) 16 vials of water for injection containing either glass particles (224 µm or 600 µm), stopper fragments, or textile fibres; (ii) five sterile injectable specialties; (iii) 20 vials of water for injection prepared under different pre-production conditions.

**Results and discussion:**

MVI and SAVI detected 100% of visible particles compared with 28% for AVI, which showed a deficiency in detecting textile fibres. All three methods correctly analysed P2S that did not contain visible particles. The three methods detected particles in vials maintained under International Organization for Standardization (ISO) 9 pre-production conditions. However, detections by (i) MVI and SAVI, and by (ii) AVI of particles contained in vials maintained under ISO 8 pre-production conditions were deemed satisfactory and unsatisfactory, respectively.

**Conclusion:**

The importance of visual inspection of P2S requires rapid, sensitive, and reliable detection methods. In this context, MVI and SAVI have proven to be more effective than AVI for a more competitive financial, training, and implementation investment.

**WHAT IS ALREADY KNOWN ON THIS TOPIC**

- Previous research has underscored the significance of visual inspection methods in ensuring the quality of compounded sterile products, yet comprehensive comparisons between manual, semi-automated, and automated methods were lacking.

**WHAT THIS STUDY ADDS**

- This study fills the gap by providing a thorough evaluation of manual, semi-automated, and automated visual inspection methods for detecting visible particles in parenteral products. It reveals the superior accuracy and efficiency of manual and semi-automated methods compared with automated inspection, particularly in detecting various types of defects.

**HOW THIS STUDY MIGHT AFFECT RESEARCH, PRACTICE OR POLICY**

- The findings highlight the importance of operator training and considerations for different production scenarios in ensuring effective particle inspection. This study’s insights can inform policy decisions regarding the selection and implementation of visual inspection methods, ultimately enhancing the safety and quality of compounded sterile products.

## Introduction

 During the COVID-19 pandemic, medicine shortages in many European Union and European Economic Area countries were highlighted.^1^ Due to the high demand for critical treatments like analgesics, sedatives, and paralytics for critically ill COVID-19 patients, various alternatives involving hospital pharmacies and flexible regulatory measures from the US Food and Drug Administration were proposed to enhance the sterile compounding and distribution of drug products.^2 3^ In hospital pharmacies, the production of sterile preparations is usually carried out in multiple small batches (< 300 units in France) of different formulations for the treatment of various diseases (online supplemental figure 1). Therefore, to ensure quality products with a low particle burden, injectable products are inspected through a life-cycle approach.^4^ Immediately after production, each unit of the batch undergoes two visual inspections by trained and qualified operators (using either manual or semi-automated inspection methods), or a single inspection through the AVI procedure, within a separate cleanroom environment (International Organization for Standardization (ISO 8), ensuring thorough quality control both immediately and for ongoing assessment. Each unit containing a defect ranging from minor to critical was rejected according to an acceptable quality limit (AQL) which defines the maximum number of defects that can be present in a batch while still being considered acceptable for release.^5^ However, ensuring the safety and quality of compounded sterile products, which are subject to less strict criteria than commercial products, requires appropriate training programmes for pharmacy technicians.^6 7^ Guidelines such as the United States Pharmacopoeia (USP) chapter 797 Pharmaceutical Compounding – Sterile Preparations aim to improve safety and reduce errors in sterile product compounding.^8^ Contamination of compounded sterile injectable drug preparations by particulate matter from microbiological, chemical, or physical sources poses serious patient safety concerns (ie, inflammatory response with granuloma formation, thrombosis), necessitating proper compounding procedures in a suitable environment.^9^ The European Pharmacopoeia (Eur. Ph.) and USP monographs require parenteral products to be practically or essentially free of visible particles, but achieving particle-free injectable preparations is practically unachievable.^10^ Quantification of sub-visible and detection of visible particles in each filled and sealed sterile preparation are mandatory, with quality control involving analytical and microbiological tests, though visible particulate matter testing presents challenges due to intrinsic and extrinsic sources of contamination.^11 12^ Regarding particulate matter in sterile products, four USP general chapters address this issue, while Eur. Ph. reports a general monograph for pharmaceutical preparations^13^ and provides recommendations on testing for particulate contamination, supplemented by two monographs.^510 14^,^18^ In the present study, our objective was to compare three methods of visual particle inspection – manual visual inspection (MVI), semi-automated visual inspection (SAVI) and automated visual inspection (AVI) – for detecting visible particles using a defect vial library examined by trained pharmacy technicians or automated inspection systems. Our study aimed to determine the origin and main sources of particulate contamination during pre-production, production, and post-production stages of compounded sterile preparations.^19^

## Materials and methods

### Manual visual inspection device

**Figure 1:**
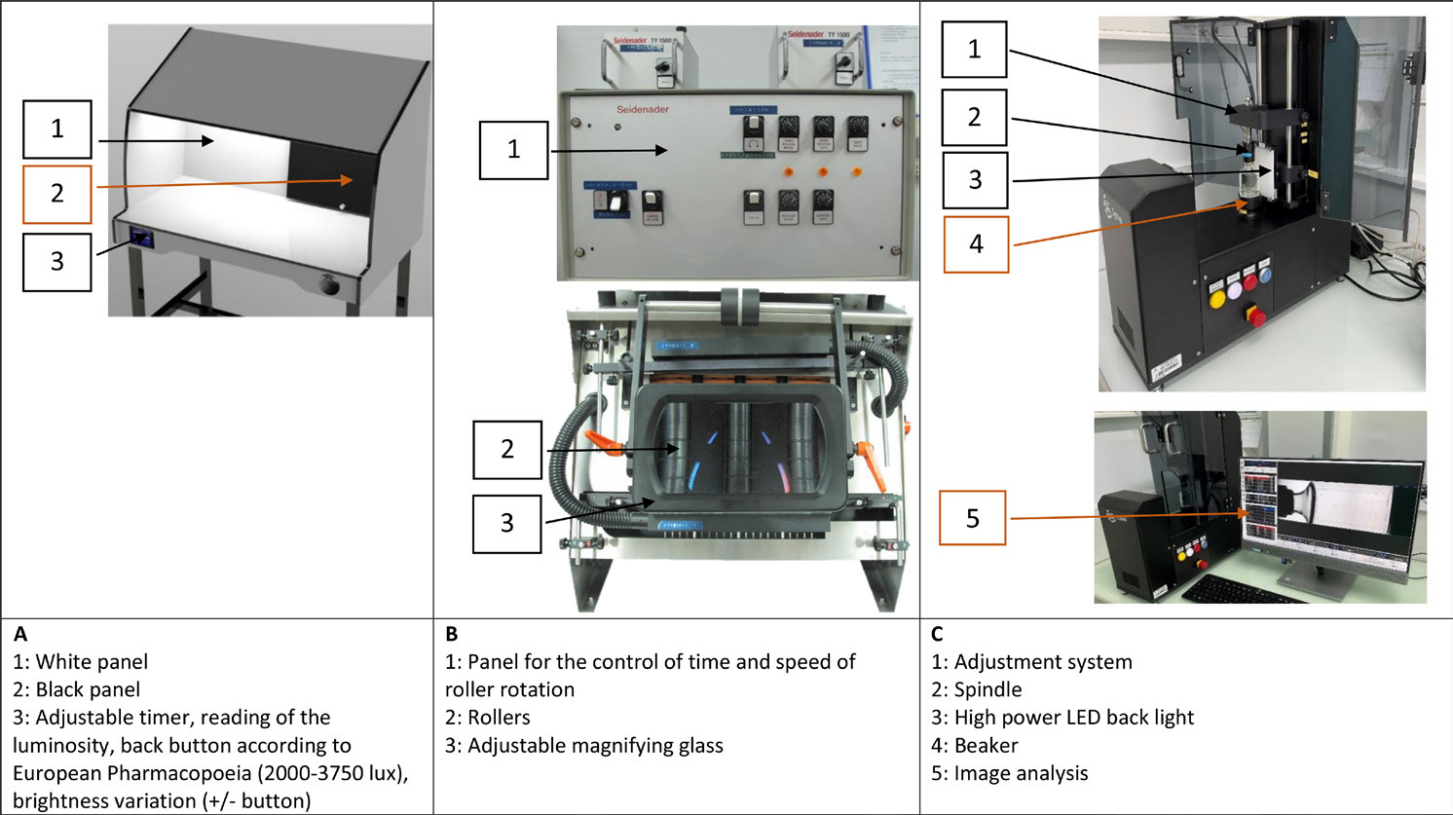
(A) manual visual inspection device (Clean View® STETDMLED12) with white and black background; (B) semi-automated visual inspection device (Seidenader® V90T) composed of a part showing the panel control, rollers and the visual field; (C) automated visual inspection device (Argo® SN 22990014 running with Gretel® software. Photos used courtesy of Fripharm®.

The MVI device used (Clean View Stetdmled12 model CVL, Sterigene, Brignais, France) featured a touch screen allowing for the adjustment of light intensities according to Eur. Ph., USP, or Japanese pharmacopoeia (figure 1A). The light intensity of this device was approximately 2350 lux, (Eur. Ph. monograph 2.9.20). Each unit underwent individual inspection against both white and black backgrounds (dimensions: width: 600 mm x depth: 200 mm x height: 200 mm), was gently agitated manually to avoid air bubble formation, and the contents were inspected for five seconds.

### Semi-automated visual inspection device

Seidenader V90T device (Siemens, Munich, Germany) allowing a fast-handling rate was used for SAVI of vials (figure 1B). Each vial was first vortexed on rollers (20 rotations for 2 seconds) then inspected under lighting (2580 lux) by an operator through a magnifying lens (x 2), for 5 seconds.

### Automated visual inspection device

**Figure 2:**
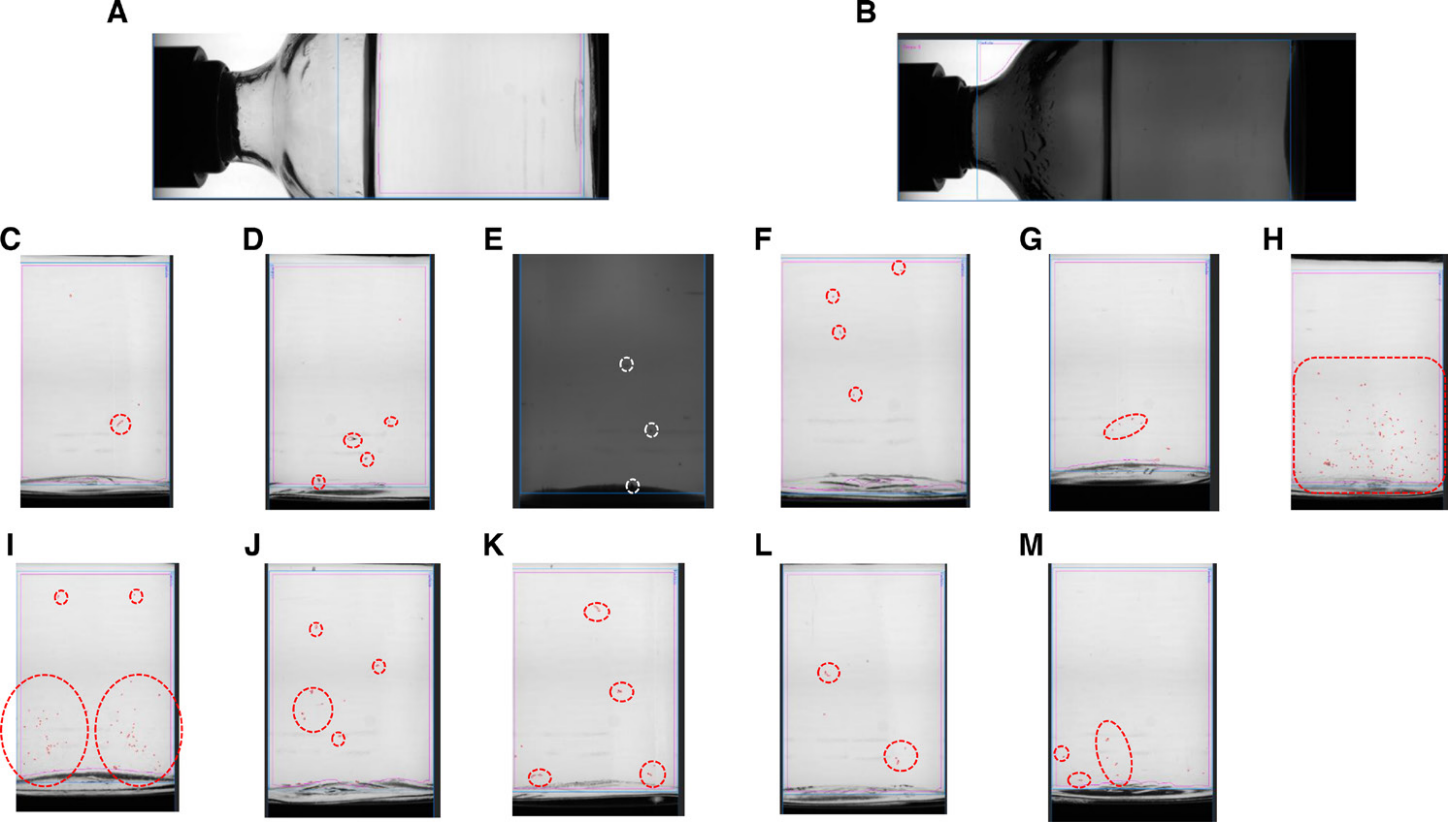
Description of different type of defects by visual inspection of transparent or amber 50-ml moulded glass vials (type I) examined by using automated visual inspection device Argo SN 22990014. No visible particle in transparent (A) and amber (B) vials. Fibres were detected in transparent vial (C). Stopper fragments were detected in transparent and amber vials (D, E), respectively. Pictures F–I show 600-µm, 280-µm, 224-µm and 50-µm glass particles, respectively while pictures J, K, depict 0.5-mm and 0.1-mm glass beads, and pictures l-M depicts 0.5-mm and 0.1-mm silica beads.

The AVI (Argo SN 22990014, Sterigene, Brignais, France) is designed to analyse individual vials after rotation (42 pulsed rotations for 2.5 seconds). The analysis is achieved by recording 40 successive computerised images obtained by using Gretel software (Optrel, Vincenza, Italy) (figure 1C). The defects were evidenced by the comparison of images showing random mobile objects on a fixed background (figure 2). In order to eliminate the artefacts from glass surface, an image analysis was performed on empty vials.^20^ The detection limit was settled to 100 µm, except for vials containing fibres in which the limit of detection was increased to 1 mm.

### Visual inspection training

Three operators were chosen based on the results of a visual acuity test to undergo further visual inspection training (VIT) (online supplemental figure 2), which they successfully completed despite the presence of astigmatism. The selection process of operators involved random selection from a pool of qualified personnel. VIT was realised by MVI of an inhouse made defect vial library composed of 41 vials (online supplemental table 1). These included: (i) 34 borosilicate glass vials (type I, Gravis, Neuville-sur-Saône, France), filled with water for injection and representative of the defects encountered in the production area including 50 mL moulded glass vials (22 transparent and four amber), eight transparent 10 mL drawn glass vials (online supplemental figure 3)^21^ and (ii) five commercial injectable defect-free vials provided by Viatris (Lyon, France) and Panpharma (Luitré-Dompierre, France) composed of moulded (2 units of 50 ml and 1 unit of 20 mL), and drawn type I glass vials (2 units of 15 mL). Two unfilled vials (one transparent 50 mL type I moulded glass vial and one transparent 10 mL type I drawn glass vial) completed the defect vial library. The operators passed the VIT test when their score was equal to or higher than 80% (ie, 33 correct detections to 41 vials), a threshold based on performance of human eye tracking.^22^

### Comparison of visual inspection methods

MVI, SAVI, and AVI methods were assessed for their reliability in detecting defects from the defect vial library. Therefore, 16 vials (eight transparent 50 mL type I moulded vials and eight transparent 10 mL type I drawn glass vials containing either 600 µm or 224 µm glass particles, stopper fragments, fibres, or defect-free) were each analysed once by three operators using MVI and SAVI methods. At the same time, 30 inspections were conducted by a single operator using the AVI method, as the inspection was automated. Furthermore, a batch of 300 compounded sterile preparations (25 mg/mL glucose - 2 mg/mL sodium chloride solution) conditioned in 50 mL type I transparent glass vials was inspected by MVI, SAVI and AVI methods.

The accuracy of methods was assessed by the calculation of analysing inspection results^23^ such as:


(Eq.1) $$\begin{array}{ll} Total\;number\;of\;inspections= & number\;of\;vials\;inspected\;\times \\ number\;of\;inspections\;\times \\ number\;of\;operators \end{array}$$

(Eq.2) $$\begin{array}{ll} Total\;inspection\;of\;defect-free\;vials= & total\;number\;of\;defect-free\;vials\;\times \\ number\;of\;inspections\;\times \\ number\;of\;operators \end{array}$$

(Eq.3) $$\begin{array}{ll} Total\;inspection\;of\;defective\;vials\;inspected= & total\;number\;of\;defective\;vials\times \\ number\;of\;inspections\;\times \\ number\;of\;operators \end{array}$$

(Eq.4) $$\text{False negative (\textbackslash{}\%) = 100 x}\frac{\text{Total number of defect-free units rated as}\text{§quot;}\text{bad}\text{§quot;}}{\text{Total number of defect-free units inspected}}$$

(Eq.5) $$\text{False positive}\text{(\textbackslash{}\%) = 100 x}\frac{\text{Total number of}\text{defective}\text{units rated as}\text{§quot;}\text{good}\text{§quot;}}{\text{Total number of defective units inspected}}$$

(Eq.6) $$\text{Unit specific error rate (\textbackslash{}\%) = 100 x}\frac{\text{Number of false positive/negative on the specific unit}}{\text{Number of inspections done on the specific unit}}$$

(Eq.7) $$\text{Overall accuracy (\textbackslash{}\%) = 100 x}\frac{\text{Total number of inspections that match the standards}}{\text{Total number of inspections}}$$

(Eq.8) $$\text{Overall error rate (\textbackslash{}\%) = 100 x}\frac{\text{Total number of inspections that do not match the standards}}{\text{Total number of inspections}}$$

Defect-free and defective vials are considered as such because visual inspection is a probabilistic process and depends on various factors (ie, environment, equipment, human, type of defect).

### Particulate contamination tracking

MVI, SAVI and AVI methods were tested for their ability to track particulate contamination in different scenarios from pre-production steps (ie, unwashed vials maintained in uncontrolled atmosphere area; washed vials maintained either in ISO 9 ambient or ISO 8–7 clean rooms; washed and depyrogenated vials maintained in ISO 8) to compounded sterile preparations (ie, 25 mg/mL glucose - 2 mg/mL sodium chloride solution; ethanol 95% and 30% solution, 5 mg/mL cisatracurium solution and 2 mg/mL pancuronium solution).^24^

In the first and second scenario, 10 unwashed (ie, worst-condition case) and 10 washed vials (five 50 mL moulded glass vials and five 10 mL type I drawn glass vials) were filled with water for injection in an ISO 9 ambient room and an ISO 8 controlled atmosphere area, respectively. In the first scenario, the filling was carried out by an operator wearing a woven cotton/polyester unsterile outfit (Klopman international, Frosinone, Italy), a mask and a hair cap without gloves while in second scenario, two operators were involved wearing an unwoven carbon/polyester sterile cleanroom outfit (Elis Cleanroom, Seyssins, France) a hair cap, a mask, overshoes, and sterile gloves. In the third scenario, the washed vials were depyrogenated then filled with water for preparation in an ISO 8 controlled atmosphere area by two operators wearing an unwoven cellulose/polyester sterile cleanroom outfit (Medline, Châteaubriant, France) a hair cap, a mask, overshoes, and sterile gloves. In the fourth scenario, the filling of washed vials by water for injection was performed in an ISO 7 controlled atmosphere area by two operators wearing an unwoven carbon/polyester sterile cleanroom outfit (Elis Cleanroom, Seyssins, France), a hair cap, a mask, overshoes, and sterile gloves followed by wrapping in polyethylene – paper autoclave steam sterilisation medical bags (SPS medical, Coulommiers, France). Thanks to at least two operators all vials were inspected by MVI, SAVI and AVI methods as described in previous sections.

Furthermore, the SAVI method (showing high speed of examination and high accuracy) was chosen to inspect several batches of compounded sterile preparations (ie, 25 mg/mL glucose - 2 mg/mL sodium chloride solution; ethanol 95% and 30% solution, 5 mg/mL cisatracurium solution and 2 mg/mL pancuronium solution) conditioned in either in-house washed, depyrogenated and sterilised 50 mL moulded glass vials or in five ready-to-use sterile and non-pyrogenic 10 mL type I drawn glass vials capped with 20 mm Raydylo capsules and a bromobutyl stopper with fluorinated polymer coating (Eurofins, Lentilly, France). All sterile preparations were produced in an ISO 5 controlled atmosphere area, with sterile vials filled by mixture filtered through sterilising filter followed by proper autoclaving for glucose, sodium chloride and ethanol solutions. The operator wore a sterile cleanroom lab coat, a mask and a hair cap, overshoes and sterile gloves. A control of sub-visible particles (10 µm and 25 µm) in mixture solution was carried out using a particle counter (PC, HACH-HIAC9703+, Beckman Coulter, Brea, CA, USA).

## Results and discussion

### Comparison of visual inspection methods

According to table 1, the overall accuracy of the MVI and SAVI methods remained consistently at 100% across 96 inspections of 16 vials. In contrast, the accuracy of the AVI method was noticeably lower, regardless of the nature of the defects, but it was influenced by the vial volume. The overall error rates for 10 mL and 50 mL vials were 75% and 22%, respectively. This finding underscores the critical role of the vortex procedure and container geometry in effectively suspending particles in the liquid during image acquisition. Notably, while all methods employ a vortex, in SAVI and MVI, operators visually observe the vortex formation, ensuring adequate particle suspension. In contrast, with AVI, if the vortex is not within the camera’s field of view, it may go undetected. This limitation could lead to incomplete particle suspension, particularly in larger vials, potentially compromising the accuracy of particle detection. Of paramount concern is the AVI’s failure to detect the liquid level in empty vials or its potential confusion between glass scratches and the actual liquid level. Additionally, it exhibited an incapacity to detect defects on the surface or wall of containers. The inability to accurately discern liquid levels in empty vials poses a significant risk to the integrity of the compounding process, casting doubt on the overall reliability and applicability of the AVI method in ensuring precise control across a spectrum of different fillings within this specific vial size range. Moreover, the AVI method exhibited a slower examination process, requiring 8 minutes for the analysis of 24 vials (MVI: 5 minutes; SAVI: 3 minutes). Furthermore, the cost-effectiveness of the MVI and SAVI methods is particularly noteworthy. These methods, requiring equipment that is significantly less expensive than that of AVI (MVI: 6 K€, SAVI: 20 K€, AVI: 88 K€), not only offer financial advantages but also demonstrate superior time efficiency. This significant time disparity raises concerns about the practicality and efficiency of the AVI method in a hospital setting.

**Table 1:** Study of the detection sensitivity of visible particles in water-containing vials with and without defects by three methods including manual, semi-automated and automated visual inspection devices

Inspection parameters	Manual		Semi-automated		Automated	
	50 mL	10 mL	50 mL	10 mL	50 mL	10 mL
Number of operators	3	3	3	3	1	1
Number of vials inspected	8	8	8	8	8	8
Number of inspections	1	1	1	1	30	30
Total number of inspections	24	24	24	24	240	240
Total inspection of defect-free vials	9	9	9	9	90	90
Total inspection of defective vials	15	15	15	15	150	150
False positive (%)	0	0	0	0	22	42
False negative (%)	0	0	0	0	23	95
Unit specific error rate (%)						
Vial #12 and #33 - *Glass particles ≥600* µm	0	0	0	0	0	100
Vial #13 and #34 - *Glass particles 224* µm	0	0	0	0	0	100
Vial #10 and #32 - *Stopper fragments*	0	0	0	0	0	100
Vial #6 and #30 - Fibres	0	0	0	0	43	83
Vial #7 and #31 - Fibres	0	0	0	0	70	90
Vial #1 and #27 No *defects*	0	0	0	0	0	0
Vial #2 and #28 No *defects*	0	0	0	0	17	53
Vial #3 and #29 No *defects*	0	0	0	0	50	73
**Overall accuracy (%**)	100	100	100	100	78	25
**Overall error rate (%**)	0	0	0	0	22	75

The study was performed on 50 mL and 10 mL type I moulded or drawn 16 glass vials, respectively.

The visual inspection of every injectable pharmaceutical within a batch is essential for identifying potential defects post-production. The MVI and SAVI methods proved effective in detecting visible particles in water within vials, whereas the AVI method failed to accurately detect the presence or absence of defects (online supplemental table 2). More precisely, MVI and SAVI were satisfying for the detection of glass particles, stopper fragments and fibres. Furthermore, the detection of fibres (ie, the most common defect encountered during production), glass and stopper fragments by the AVI method showed a major error rate (72%: 86 errors /120 fibre analyses and 50%: 90 errors/180 glass particles and stopper fragment analyses) likely due to the particular geometry (length~5 mm; low refractive index) of the contaminant.

This emphasised the intricate challenges associated with the design, implementation, and maintenance of the AVI method.^25^ The fluctuating accuracy of AVI in the absence of defects (32% overall error rate; 58 errors/180 analyses of six vials (#1, #27, #2, #28, #3, #29)) contrasted with its correct detection of defects in five commercial injectable particle-free vials from industrial pharmaceutical production, subject to stringent control requirements referenced in Current Good Manufacturing Practice (cGMP) regulations.^26^ Similar outcomes were identified in the inspection of compounding sterile preparations, specifically a glucose solution (25 mg/mL) - sodium chloride (2 mg/mL), using MVI, SAVI, and AVI methods (online supplemental table 3). An analysis of images generated by the reviewing software revealed that the observed defects were concentrated in the vial’s meniscus, potentially causing confusion between air bubbles and actual particles, overestimating particle counting. Thus, the observed particle rates detected by the MVI and SAVI methods were comparable, resulting in rejection rates of 4% and 5% of vials, respectively. Therefore, considering the limitations of the AVI method, it is highly advisable to complementarily employ either the MVI or SAVI method for the detection of particles that could potentially lead to various vascular and inflammatory side-effects. This dual control mechanism was deemed crucial from both economic and patient health perspectives.^27 28^

### Particulate contamination tracking

The influence of different operating conditions on particulate contamination in 50 mL and 10 mL vials is documented in tables2  3, respectively. Under the most unfavourable operating conditions (ie, ISO 9 room area; unsterile regular coat without gloves), all detection methods identified defects in every vial. However, under other operating conditions, MVI and SAVI methods exhibited similar detection capabilities, while the AVI method displayed inconsistencies in detection. Clearly, the primary sources of particulate contamination were associated with the depyrogenation process of the vials into the oven, followed by polyethylene-paper wrapping. These findings were substantiated by the low levels of defects detected in ready-to-use 50 mL and 10 mL vials filled with cisatracurium and pancuronium solutions, as opposed to the higher particulate levels observed in lab-washed and depyrogenated vials filled with ethanol and glucose saline solutions (table 4). The extent of contamination was not attributed to intrinsic contamination caused by a decrease in solute solubility, as suggested by the low sub-visible particle counting. Additionally, in Eur. Ph. monograph,^15^ thresholds for counting non-visible particles vary depending on particle size and container volume. For instance, for particles sized between 10 and 25 micrometres, the threshold is typically 6000 particles per container, while for smaller particles, it may be 25 000 particles per container. More likely, it was assumed that extensive human handling, along with the washing and depyrogenation processes, contributed by e.g. glass abrasion to an increased introduction of particle contaminants into the vials.^29^ Earlier studies showed that the detectability of certain process-related particles (eg, fibres, particles) in compounding sterile preparations was highly dependent not only on the trained personnel involved^30^ but also on the type and morphology of the defect, and the geometry of the empty and filled container.^31^ Taken together these results showed that the particle control is part of a global strategy for limiting defects through the use of quality containers with effective washing, depyrogenation and sterilisation procedures limiting the introduction of particles emitted by personnel in a controlled environment.

**Table 2:** Study of the detection of visible particles in 50 mL type I moulded glass vials by three methods including manual, semi-automated performed by two operators (#1, #2) and automated visual inspection devices.

Operating condition	Vials	Visible particles inspection				
		Manual		Semi-automated		Automated
		#1	#2	#1	#2	
No cleanroom ISO 9 Woven cotton/polyester unsterile outfit without gloves	Unwashed and unwrapped					
	Vial 1.1 to 1.5	Bad	Bad	Bad	Bad	5 Bad – 0 Good
Cleanroom D ISO 8 Unwoven carbon/polyester sterile cleanroom outfit with sterile gloves	Washed and unwrapped					
	Vial 2.1	Good	Good	Good	Good	5 Good – 0 Bad
	Vial 2.2	Good	Good	Good	Good	5 Good – 0 Bad
	Vial 2.3	Bad	Bad	Bad	Bad	5 Bad – 0 Good
	Vial 2.4	Bad	Bad	Bad	Bad	2 Bad – 3 Good
	Vial 2.5	Bad	Bad	Bad	Bad	1 Bad – 4 Good
Cleanroom D ISO 8 Unwoven cellulose/polyester sterile cleanroom outfit with sterile gloves	Washed, depyrogenated and unwrapped					
	Vial 3.1	Good	Good	Good	Good	3 Good – 2 Bad
	Vial 3.2	Bad	Bad	Bad	Bad	5 Bad – 0 Good
	Vial 3.3	Bad	Bad	Bad	Bad	5 Bad – 0 Good
	Vial 3.4	Bad	Bad	Bad	Bad	4 Bad – 1 Good
	Vial 3.5	Bad	Bad	Bad	Bad	4 Bad – 1 Good
Cleanroom C ISO 7 Unwoven carbon/polyester sterile cleanroom outfit with sterile gloves	Washed and wrapped					
	Vial 4.1	Good	Good	Good	Good	4 Good – 1 Bad
	Vial 4.2	Good	Good	Good	Good	3 Good – 2 Bad
	Vial 4.3	Bad	Bad	Bad	Bad	5 Bad – 0 Good
	Vial 4.4	Bad	Bad	Bad	Bad	2 Bad – 3 Good
	Vial 4.5	Bad	Bad	Bad	Bad	2 Bad – 3 Good

Good: No defect detected.

Bad: One or more defects detected.

For the washed vials, one set was bagged, the other was filled directly at the end of washing. The vials were successfully inspected five times by an automated visual inspection device.

**Table 3:** Study of the detection of visible particles in 10 mL type I moulded glass vials by three methods including manual, semi-automated performed by two operators (#1, #2) and automated visual inspection devices

Operating condition	Vials	Visible particles inspection				
		Manual		Semi-automated		Automated
		#1	#2	#1	#2	
No cleanroom ISO 9 Woven cotton/polyester unsterile outfit without gloves	Unwashed and unwrapped					
	Vial 5.1 to 5.5	Bad	Bad	Bad	Bad	5 Bad – 0 Good
Cleanroom D ISO 8 Unwoven carbon/polyester sterile cleanroom outfit with sterile gloves	Washed and unwrapped					
	Vial 6.1	Good	Good	Good	Good	5 Good – 0 Bad
	Vial 6.2	Good	Good	Good	Good	5 Good – 0 Bad
	Vial 6.3	Good	Good	Good	Good	5 Good – 0 Bad
	Vial 6.4	Good	Good	Good	Good	5 Good – 0 Bad
	Vial 6.5	Bad	Bad	Bad	Bad	5 Bad – 0 Good
Cleanroom D ISO 8 Unwoven cellulose/polyester sterile cleanroom outfit with sterile gloves	Washed, depyrogenated and unwrapped					
	Vial 7.1	Good	Good	Good	Good	5 Good – 0 Bad
	Vial 7.2	Good	Good	Good	Good	5 Good – 0 Bad
	Vial 7.3	Good	Good	Good	Good	1 Good – 4 Bad
	Vial 7.4	Bad	Bad	Bad	Bad	5 Bad – 0 Good
	Vial 7.5	Bad	Bad	Bad	Bad	5 Bad – 0 Good
Cleanroom C ISO 7 Unwoven carbon/polyester sterile cleanroom outfit with sterile gloves	Washed and wrapped					
	Vial 8.1	Good	Good	Good	Good	5 Good – 0 Bad
	Vial 8.2	Good	Good	Good	Good	4 Good – 1 Bad
	Vial 8.3	Bad	Bad	Bad	Bad	5 Bad – 0 Good
	Vial 8.4	Bad	Bad	Bad	Bad	5 Bad – 0 Good
	Vial 8.5	Bad	Bad	Bad	Bad	2 Bad – 3 Good

Good: No defect detected.

Bad: One or more defects detected.

For the washed vials, one set was bagged, the other was filled directly at the end of washing. The vials were successfully inspected five times by an automated visual inspection device.

**Table 4:** Study of the detection of visible defects by a semi-automated visual inspection device and detection of particles by particle counting following the analysis of five production lots of 50 mL and 10 mL

Compounded sterile preparations	Ethanol 95%	Ethanol 30%	AP ISO	Cisatracurium besilate 5 mg/mL	Pancuronium bromide 2 mg/mL
Batch					
Volume of unit (ml)	50	10	50	50	10
Number of units	229	288	300	309	544
Visible defects >50 µm					
Fibres	40 (17.4%)	8 (2.8%)	7 (2.3%)	2 (0.6%)	7 (1.3%)
Particles	21 (9.2%)	7 (2.4%)	4 (1.3%)	2 (0.6%)	5 (0.9%)
Altered packaging	0	0	3 (1%)	0	3 (0.5%)
Total	61 (26.6%)	15 (5.2%)	14 (4.6%)	4 (1.2%)	15 (2.7%)
Sub-visible particles					
10 µm (< 6 000 particles/vial)	17	139	45	105	38
25 µm (< 600 particles/vial)	1	13	1	65	0

AP ISO is a mixture of glucose 25 mg/mL and sodium chloride 2 mg/mL used as neonatal rehydration solution.

The term “altered packaging” encompasses a range of defects, including broken vials or lifted caps.

Although previous reports focused on the threshold for visibility of defects^32^ (online supplemental figure 4), in the present study, the detectability of large defects (eg, glass particles ≥600 µm, fibres) presenting potentially high or very weak density (increasing the probability of fast sedimentation or buoyancy) was assessed by visual and automatic inspection. Surprisingly, the detectability of large defects by AVI was strongly influenced by the volume of the dispersing medium in containers; the lower the dispersing volume, the poorer the detectability. Large particulate matter has a higher likelihood of causing harm to patients if present in injectable medications. Therefore, the defect library has to include not only the potential defects encountered in vials but also various dispersing volumes to properly qualify both the method of inspection and the operators. Additionally, apart from particulate contamination, compounding sterile preparations entails other types of risks, including bacterial, viral, or fungal contamination. These risks emphasise the importance of stringent terminal sterilisation procedures to ensure the safety and efficacy of compounded products. Thus, comprehensive quality control measures encompass not only particulate monitoring but also microbial testing and sterilisation validation protocols to mitigate these risks effectively.

## Conclusion

In conclusion, our analysis of a defect vial library and various operating scenarios revealed significant differences in method performance and efficiency. MVI and SAVI consistently demonstrated high accuracy. The study underscored the critical role of human involvement in visual inspection and identified challenges in AVI’s ability to handle diverse types of defects. Additionally, our research highlighted difficulties in tracking particulate contamination across different production stages, uncovering potential sources of contamination such as washing, depyrogenation, and environmental factors affecting vial maintenance.

In future perspectives, optimising AVI for a full vial scan, rather than center-focused inspection, could improve defect detection and reduce false negatives. Addressing challenges related to container geometry and dispersant volume is essential. Limitations observed in our AVI setup are intrinsic to the technology, necessitating advanced algorithms and hardware. Emerging technologies like TURBISCAN, employing SMLS, may complement AVI, enhancing pharmaceutical quality control.

## Supplementary material

10.1136/ejhpharm-2024-004143 online supplemental file 1

10.1136/ejhpharm-2024-004143 online supplemental file 2

## Data Availability

No data are available.
