# Supplementary material for: Comparing visual inspection methods for parenteral products in hospital pharmacy: between reliability, cost, and operator formation considerations
Source: Eur J Hosp Pharm. 2024 May 24;32(6):e004143. doi: 10.1136/ejhpharm-2024-004143 (PMC12573397; doi:10.1136/ejhpharm-2024-004143)
Supplement: online supplemental file 1 [file ejhpharm-32-6-s001.pdf]

Legends of figures

Supplemental figure 1. Flow chart of injectable products production.
Supplemental figure 2. Result of the visual acuity test of the three operators.
Supplemental figure 3. Pictures and drawings of 50-ml and 10-ml type I transparent glass molded vials (A1, A2, B1, B2) associated with their 20-mm stoppers(C1) and caps (C2, C3) from Gravis® supplier.
Supplemental figure 4. Size range of particles as specified in USP <1788>. The visible gray zone represents the size of particles with a limited detection probability for human vision.

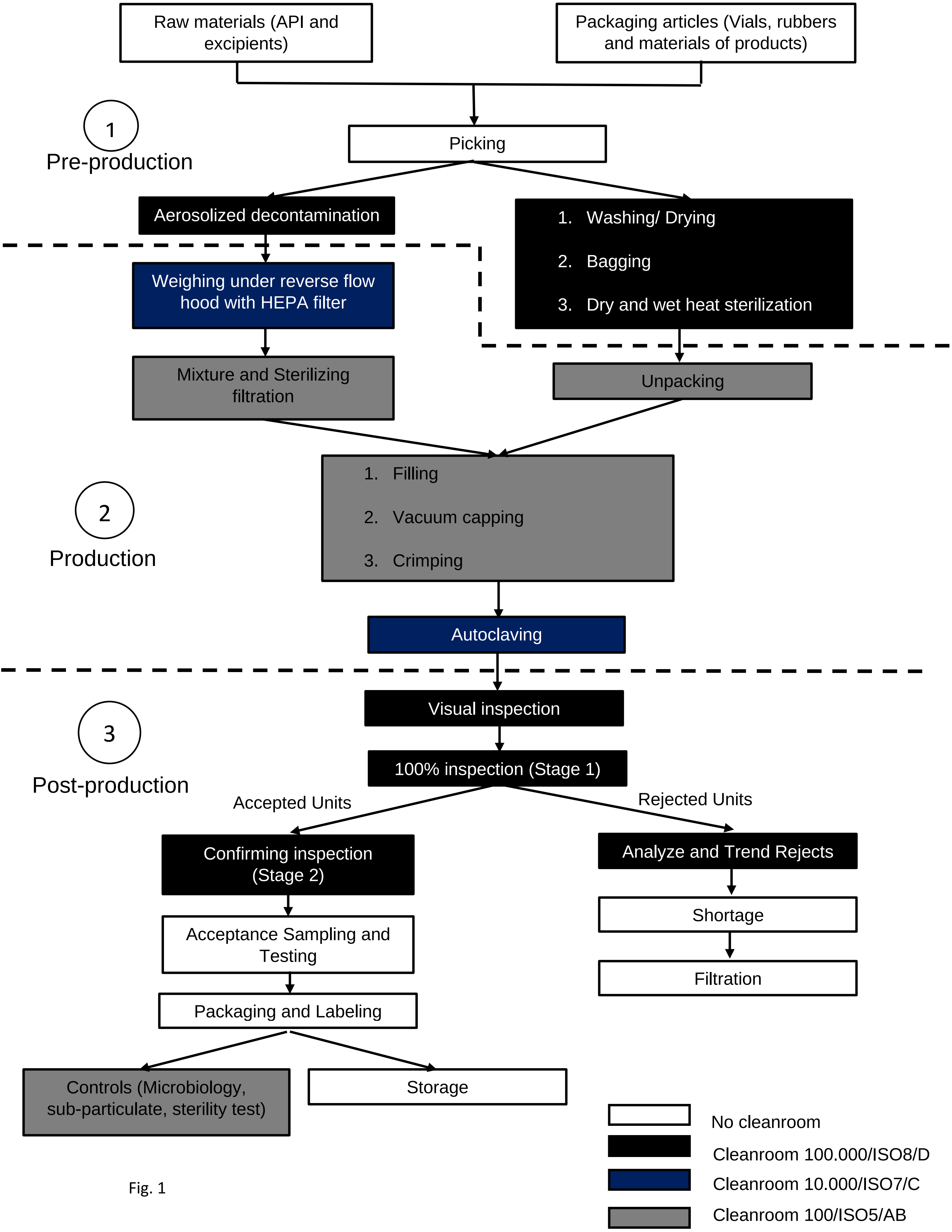

Raw materials (API and excipients)
Packaging articles (Vials, rubbers and materials of products)
1
Pre-production
Picking
Aerosolized decontamination
1. Washing/ Drying
2. Bagging
3. Dry and wet heat sterilization
Weighing under reverse flow hood with HEPA filter
Mixture and Sterilizing filtration
Unpacking
2
Production
1. Filling
2. Vacuum capping
3. Crimping
Autoclaving
3
Post-production
Visual inspection
100% inspection (Stage 1)
Accepted Units
Rejected Units
Confirming inspection (Stage 2)
Analyze and Trend Rejects
Acceptance Sampling and Testing
Shortage
Filtration
Packaging and Labeling
Controls (Microbiology, sub-particulate, sterility test)
Storage
No cleanroom
Cleanroom 100.000/ISO8/D
Cleanroom 10.000/ISO7/C
Cleanroom 100/ISO5/AB

Fig. 1

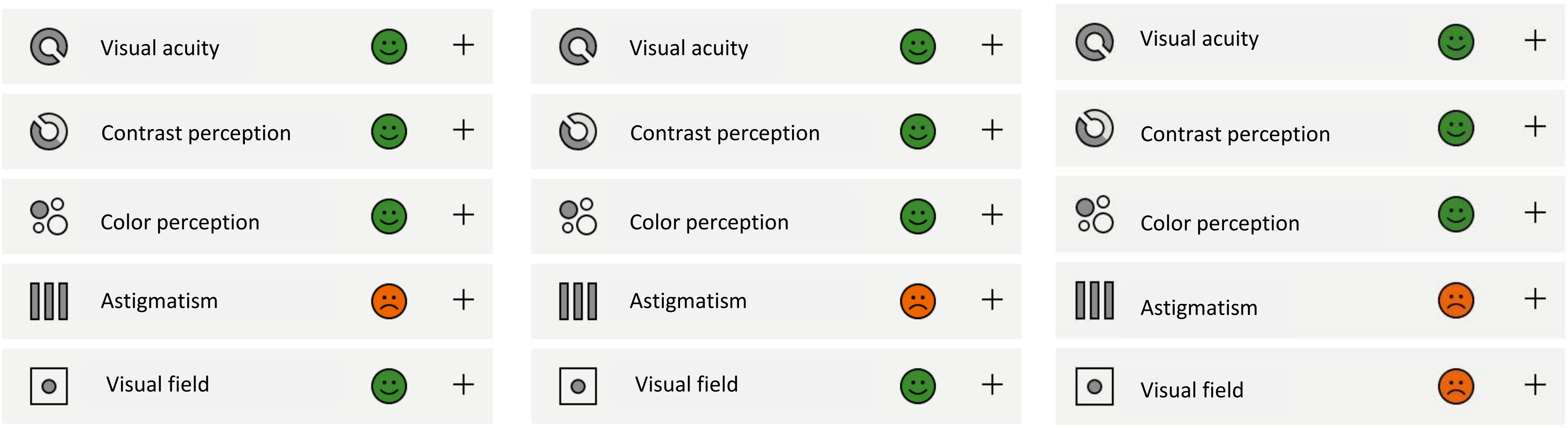

Operator 1 Operator 2 Operator 3

Fig.2

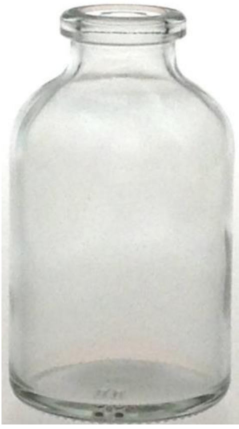

A1

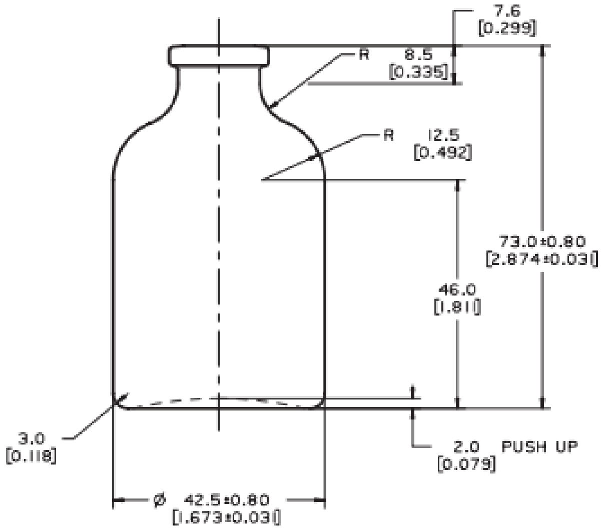

A2

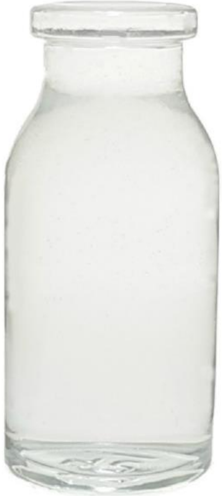

B1

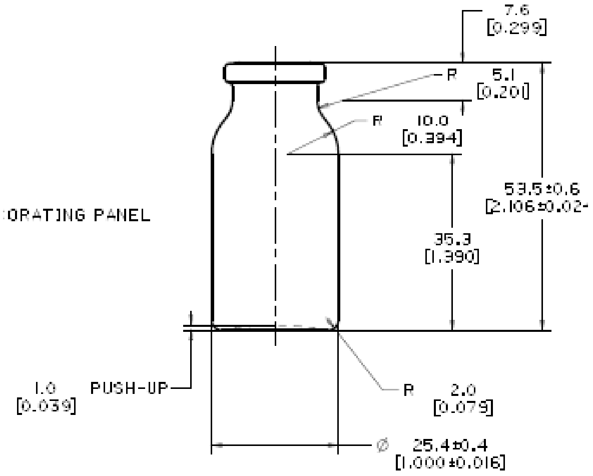

B2

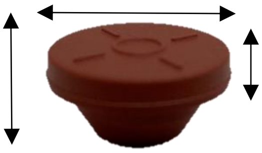

19 mm x 0.3 mm x 10 mm

C1

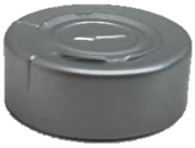

C2

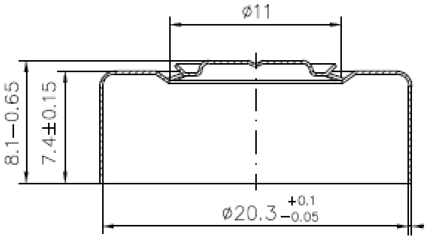

C3

Fig.3

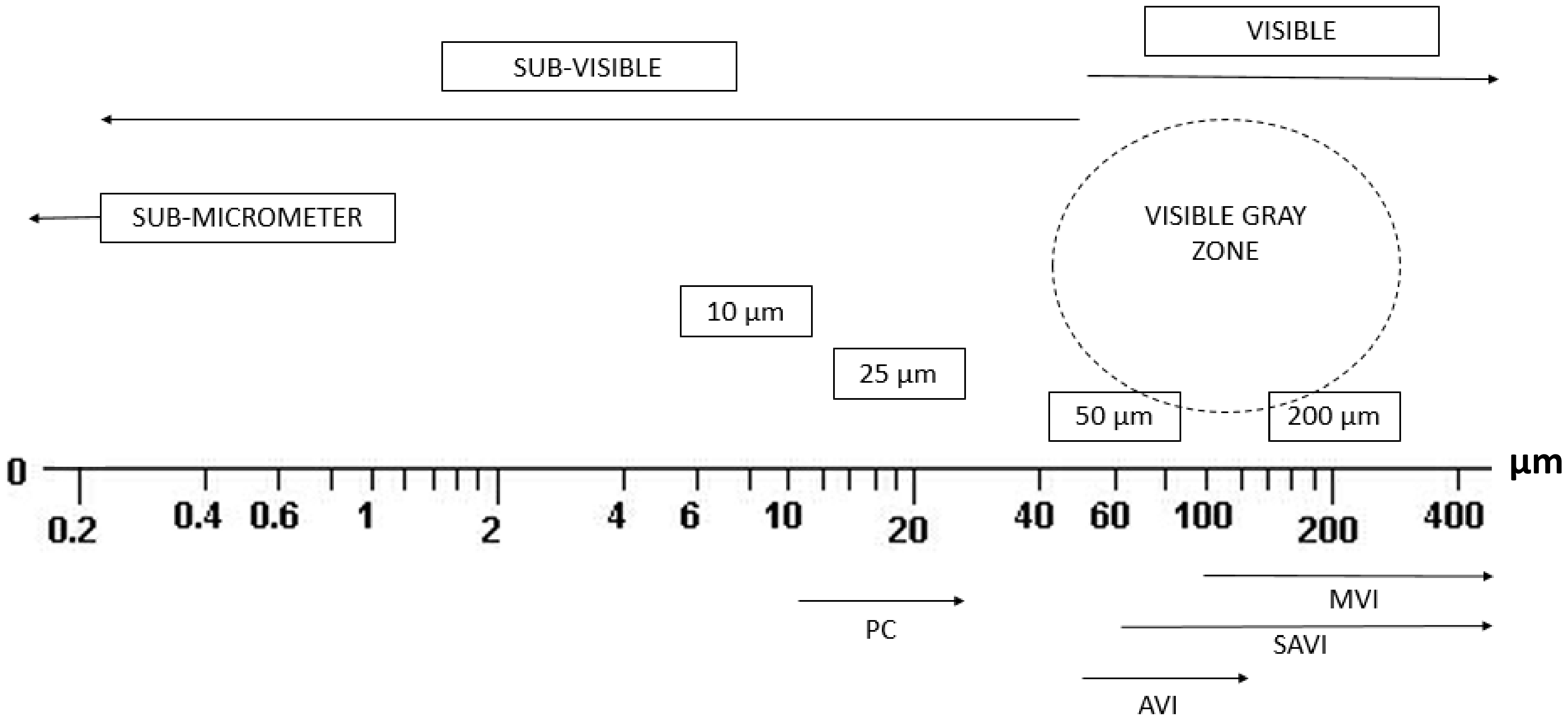
VISIBLE
SUB-VISIBLE
SUB-MICROMETER
VISIBLE GRAY ZONE
10 µm
25 µm
50 µm
200 µm
0
µm
0.2
0.4
0.6
1
2
4
6
10
20
40
60
100
200
400
MVI
PC
SAVI
AVI

Fig.4
