# Supplementary material for: Comparing visual inspection methods for parenteral products in hospital pharmacy: between reliability, cost, and operator formation considerations
Source: Eur J Hosp Pharm. 2024 May 24;32(6):e004143. doi: 10.1136/ejhpharm-2024-004143 (PMC12573397; doi:10.1136/ejhpharm-2024-004143)
Supplement: online supplemental file 2 [file ejhpharm-32-6-s002.pdf]

Supplemental table 1. Composition of 34 borosilicate glass vials (type I, Gravis®, Neuville-sur-Saône, France), filled with water for injection and representative of the defects encountered in the production area including 22 transparent, 4 amber 50-ml molded glass vials, 8 transparent 10-mL drawn glass vials.

Vials number (1-41)	Defect
50-mL transparent (1-22) vials	
#1 - #5	Exempt of particles
#6 - #9	Fibers
#10 - #11	Stopper fragments
#12	Glass particles ≥ 600 µm
#13	Glass particles 224 µm
#14	Glass particles 280 µm
#15	Glass particles 50 µm
#16	Glass beads 1 mm
#17	Glass beads 0.5 mm
#18	Glass beads 0.1 mm
#19	Silica beads 0.5 mm
#20	Silica beads 0.1 mm
#21 - #22	Particles
50-ml amber (23-26) vials	
#23 - #24	Exempt of particles
#25	Fibers
#26	Stopper fragments
10-ml transparent (27-34) vials	
#27 - #29	Exempt of particles
#30 - #31	Fibers
#32	Stopper fragments
#33	Glass particles ≥ 600 µm
#34	Glass particles 224 µm
Commercial injectable vials (#35-#39) vials	Exempt of particles
Unfilled vials (#40-#41)	Exempt of particles and liquid

Supplemental table 2. Study of the detection of visible particles in water containing vials with and without defects by three methods including manual, semi-automated and automated visual inspection devices.

Defect vial library	Vials	Visible particles inspection						
		Manual			Semi-automated			Automated
		#1	#2	#3	#1	#2	#3	
Glass particles ≥ 600 µm	Vial #12 $_{50\ mL}$	Bad	Bad	Bad	Bad	Bad	Bad	30 Bad – 0 Good
	Vial #33 $_{10\ mL}$	Bad	Bad	Bad	Bad	Bad	Bad	0 Bad – 30 Good
Glass particles 224 µm	Vial #13 $_{50\ mL}$	Bad	Bad	Bad	Bad	Bad	Bad	30 Bad – 0 Good
	Vial #34 $_{10\ mL}$	Bad	Bad	Bad	Bad	Bad	Bad	0 Bad – 30 Good
Stopper fragments	Vial #10 $_{50\ mL}$	Bad	Bad	Bad	Bad	Bad	Bad	30 Bad – 0 Good
	Vial #32 $_{10\ mL}$	Bad	Bad	Bad	Bad	Bad	Bad	0 Bad – 30 Good
Fibers	Vial #6 $_{50\ mL}$	Bad	Bad	Bad	Bad	Bad	Bad	17 Bad – 13 Good
	Vial #30 $_{10\ mL}$	Bad	Bad	Bad	Bad	Bad	Bad	5 Bad – 25 Good
	Vial #7 $_{50\ mL}$	Bad	Bad	Bad	Bad	Bad	Bad	9 Bad – 21 Good
	Vial #31 $_{10\ mL}$	Bad	Bad	Bad	Bad	Bad	Bad	3 Bad – 27 Good
No defects	Vial #1 $_{50\ mL}$	Good	Good	Good	Good	Good	Good	30 Good – 0 Bad
	Vial #27 $_{10\ mL}$	Good	Good	Good	Good	Good	Good	30 Good – 0 Bad
	Vial #2 $_{50\ mL}$	Good	Good	Good	Good	Good	Good	25 Good – 5 Bad
	Vial #28 $_{10\ mL}$	Good	Good	Good	Good	Good	Good	14 Good – 16 Bad
	Vial #3 $_{50\ mL}$	Good	Good	Good	Good	Good	Good	15 Good – 15 Bad
	Vial #29 $_{10\ mL}$	Good	Good	Good	Good	Good	Good	8 Good – 22 Bad

Good: No defect detected
Bad: One or more defects detected
This detection of defect was performed by three operators (#1, #2, #3) on 50-ml and 10-ml type I molded or drawn 16 glass vials (i.e, 96 inspections) respectively. Furthermore, 16 vials were successively inspected thirty times by an automated visual inspection device (i.e, 480 inspections).

Supplemental table 3. Study of the detection of visible defects by three visual inspection devices: manual, semi-automated and automated following the analysis of AP ISO production lots of 50 ml.

**Hospital preparation**		**AP ISO**	
**Batch**			
Volume of unit (ml)		50	
Number of units		299	
**Visible defects > 50 µm**	Manual	Semi-automated	Automated
Fibers	5 (2%)	6 (2%)	2 (1%)
Particles	6 (2%)	8 (3%)	35 (12%)
Altered packaging	0	0	0
Total	11 (4%)	14 (5%)	37 (13%)

AP ISO is a mixture of glucose (25 mg/ml) and sodium chloride (2 mg/ml) used as neonatal rehydration solution.

The term "altered packaging" encompasses a range of defects, including broken vials or lifted caps.
